# The role of tumor microenvironment reprogramming in primary liver cancer chemotherapy resistance

**DOI:** 10.3389/fonc.2022.1008902

**Published:** 2022-11-24

**Authors:** Chunyu Zhao, Shanshuo Liu, Feng Gao, Yawen Zou, Zhigang Ren, Zujiang Yu

**Affiliations:** ^1^ Department of Infectious Diseases, The First Affiliated Hospital of Zhengzhou University, Zhengzhou, China; ^2^ Jinan Microecological Biomedicine Shandong Laboratory, Jinan, Shandong, China; ^3^ Gene Hospital of Henan Province; Precision Medicine Center, The First Affiliated Hospital of Zhengzhou University, Zhengzhou, China

**Keywords:** tumor microenvironment, reprogramming, primary liver cancer, chemotherapy resistance, hepatocellular carcinoma

## Abstract

Primary liver cancer (PLC), including hepatocellular carcinoma (HCC) and intrahepatic cholangiocarcinoma (ICC), and other rare tumours, is the second leading cause of cancer-related mortality. It has been a major contributor to the cancer burden worldwide. Of all primary liver cancer, HCC is the most common type. Over the past few decades, chemotherapy, immunotherapy and other therapies have been identified as applicable to the treatment of HCC. However, evidence suggests that chemotherapy resistance is associated with higher mortality rates in liver cancer. The tumour microenvironment (TME), which includes molecular, cellular, extracellular matrix(ECM), and vascular signalling pathways, is a complex ecosystem. It is now increasingly recognized that the tumour microenvironment plays a pivotal role in PLC prognosis, progression and treatment response. Cancer cells reprogram the tumour microenvironment to develop resistance to chemotherapy drugs distinct from normal differentiated tissues. Chemotherapy resistance mechanisms are reshaped during TME reprogramming. For this reason, TME reprogramming can provide a powerful tool to understand better both cancer-fate processes and regenerative, with the potential to develop a new treatment. This review discusses the recent progress of tumour drug resistance, particularly tumour microenvironment reprogramming in tumour chemotherapy resistance, and focuses on its potential application prospects.

## Introduction

Over the past few years, cancer has always been a significant public health problem worldwide. According to the data the American Cancer Society reported in 2021, there are nearly 1.9 million new cancer cases and more than 600 thousand cancer deaths in the United States (1). Of these, liver cancer is the most common cause of cancer death worldwide and one of the fifth most common cancers in the United States, with its incidence rising yearly (2). The occurrence of PLC is mainly associated with chronic hepatitis B virus (HBV) and hepatitis C virus (HCV) infections. However, other factors have also been implicated in the occurrence of HCC, including fatty liver disease, dietary aflatoxin exposure, alcohol-related cirrhosis, smoking, obesity, fatty liver disease, iron overload, Mellitus-related non-alcoholic fatty liver disease and type 2 diabetes (3, 4). Although ICC accounts for only a small proportion of primary liver cancer in most parts of the world, it is the most common subtype of primary liver cancer in Thailand due to the high incidence of chronic liver fluke infection. Cirrhosis is also one of its risk factors (5, 6).

The treatment of HCC has dramatically improved over the past decades. The Barcelona Clinic Liver Cancer (BCLC) staging system is the main basis for treatment. In principle, patients with early-stage are suitable for surgical resection, liver transplantation, and local ablation, while TACE is the first choice for patients with intermediate HCC. Due to the lack of effective chemoprevention strategies and early diagnosis, most patients are found at an advanced stage. However, the late prognosis is poor, and only systemic therapy can prolong survival time with a median survival time for advanced HCC of ~6 months (7). Sorafenib was the only systemic therapy approved for patients with advanced tumors in the last dozen years (8). The results of a global open-label randomized Phase-III trial (REFLECT) demonstrated that lenvatinib improved the overall survival of patients with advanced HCC, the first new drug to be approved in the first-line setting for advanced-stage HCC in more than 10 years, which represented a breakthrough in the clinical management of this cancer (7). Despite this, in advanced liver cancer, the survival benefit of these drugs is limited (9, 10). Despite this, in advanced liver cancer, the survival benefit of these drugs is limited.

The environment in which HCC tumor cells grow is called the liver tumor microenvironment, which is a complex mixture of tumor cells and stromal cells and the proteins they secrete (11). Normally, the stroma maintains the physiological homeostasis of normal tissues, and some stromal components act as a physical barrier to tumor formation (12, 13). However, neoplastic cells cause various changes, and stroma is inappropriately activated in cancer, transforming adjacent TME into pathological entities to support cancer development and make a contribution to the malignant characteristics of tumor cells (14). The elements of a typical TME are made up of surrounding blood vessels, cancer-associated fibroblasts(CAFs), immune and inflammatory cells, cytokines, chemokines or enzymes, and extracellular matrix (ECM) (15). There is increasing evidence that liver cancer progression and metastasis are influenced by the tumor microenvironment.

An adverse tumor microenvironment in a single tumor is recognized as common in most solid malignancies. It has been found that the degree of heterogeneity in the tumor microenvironment of normal and malignant cells is negatively correlated with the prognosis of patients (16). Some of the harmful features of the tumor microenvironment through reprogramming can act alone or in combination with cancer progression(via e.g. resistance to apoptosis, promotion of genetic instability and mutation, continuous angiogenesis, and distant metastasis), leading to chemotherapeutic resistance and ultimately poor patient outcomes (17). In this review, we mainly focus on the mechanism of tumor microenvironment reprogramming on chemotherapy resistance in PLC to spark new ideas for designing more specific therapies for cancer.

## Cancer-associated fibroblasts

Among all the stromal cells that make up the tumor microenvironment, CAFs are the most abundant tumor stromal cell type and a key element of TME (18). CAFs predominantly arise from tissue-resident fibroblasts and mesenchymal stem cells (19–22). But studies have shown that even under limited conditions. Transformation of adipocytes, pericytes, and endothelial cells has also been observed (23). Normally, fibroblasts are usually quiescent, but fibroblasts can be activated under certain conditions, such as wound healing response, tissue fibrosis acute and chronic inflammation (24–26). Tumor cells recruit and secrete growth factors such as transforming growth factor β (TGFβ), platelet-derived growth factor (PDGF), and interleukin-6 (IL-6) to stimulate fibroblasts to convert to CAFs (23, 27). Although α-SMA, FAP, and PDGF receptor-α (PDGFRα)can recognize activated fibroblasts, there are still no comprehensive and specific biomarkers (23, 28, 29). Numerous studies have suggested that CAFs are associated with treatment resistance to colorectal cancer, ovarian cancer, breast cancer, and stomach cancer (30–33). Cancer-associated fibroblasts can mediate drug resistance by reprogramming the metabolic process of tumor cells (34).

Metastasis and metabolic reprogramming are known as two major features of cancer (35). The reason why liver tumorigenesis or tumor progression inevitably leads to metabolic reprogramming is that life is not only the largest metabolic organ in the human body but also is associated with almost all central metabolic processes. Tumor cells undergo metabolic reprogramming to meet bioenergy and biosynthesis requirements to maintain their abnormal proliferation and adapt to the tumor microenvironment (36). Unlike normal cells, tumor cells rely on glycolysis to produce energy even under aerobic conditions, also known as the Warburg effect (37). In this process, the rate of glucose uptake dramatically increases and produces more lactate. Cancer-associated fibroblasts have been observed to influence cancer drug response and play a positive metabolic role in tyrosine kinase inhibitor (TKIs) resistance. Specifically, increased lactate secretion leads to upregulation of hepatocyte growth factor (HGF) production by cancer-associated fibroblasts in a nuclear factor KB-dependent manner. MET-dependent signal transduction in cancer is activated by increased HGF and endows sustained resistance to TKIs (38). Studies have shown that in HCC patients with high c-MET expression, HGF reduces the anti-proliferation, pro-apoptotic and anti-invasion effects of lenvatinib on HCC cells. In addition, the activation of the HGF/c−MET axis promotes lenvatinib resistance in hepatocellular carcinoma cells (39). Moreover, after long-term sorafenib treatment, HGF upregulation induces the autocrine activation of the HGF ⁄c-Met signaling pathway, increasing the anti-apoptotic and invasion ability of HCC cells, and leading to resistance to sorafenib (40). It can be seen that the HGF/c-Met axis plays an important role in the chemotherapy resistance of liver cancer. Several HGF/c-MET inhibitor drugs have been e assessed in clinical trials (**Table 1**).

**Table 1:** Clinical trials of some HGF/c-Met signaling inhibitors in hepatocellular carcinoma patients.

Drug	Targets of inhibitor	Phase	Activity	Status
Tivantinib	c-Met	III	Failed	Completed
Cabozantinib	c-Met, VEGFRs, RET, KIT and AXL	III	Anti-tumor	Completed
Foretinib	c-Met, AXL, RON, VEGFR2 and TIE-2	II	Anti-tumor	Completed
Capmatinib	c-Met	II	Anti-tumor	Active, not recruiting
Tepotinib	c-Met	II	Anti-tumor	Completed
Golvatinib	c-Met, VEGFR-2	II	Anti-tumor	Completed

**Figure 1:**
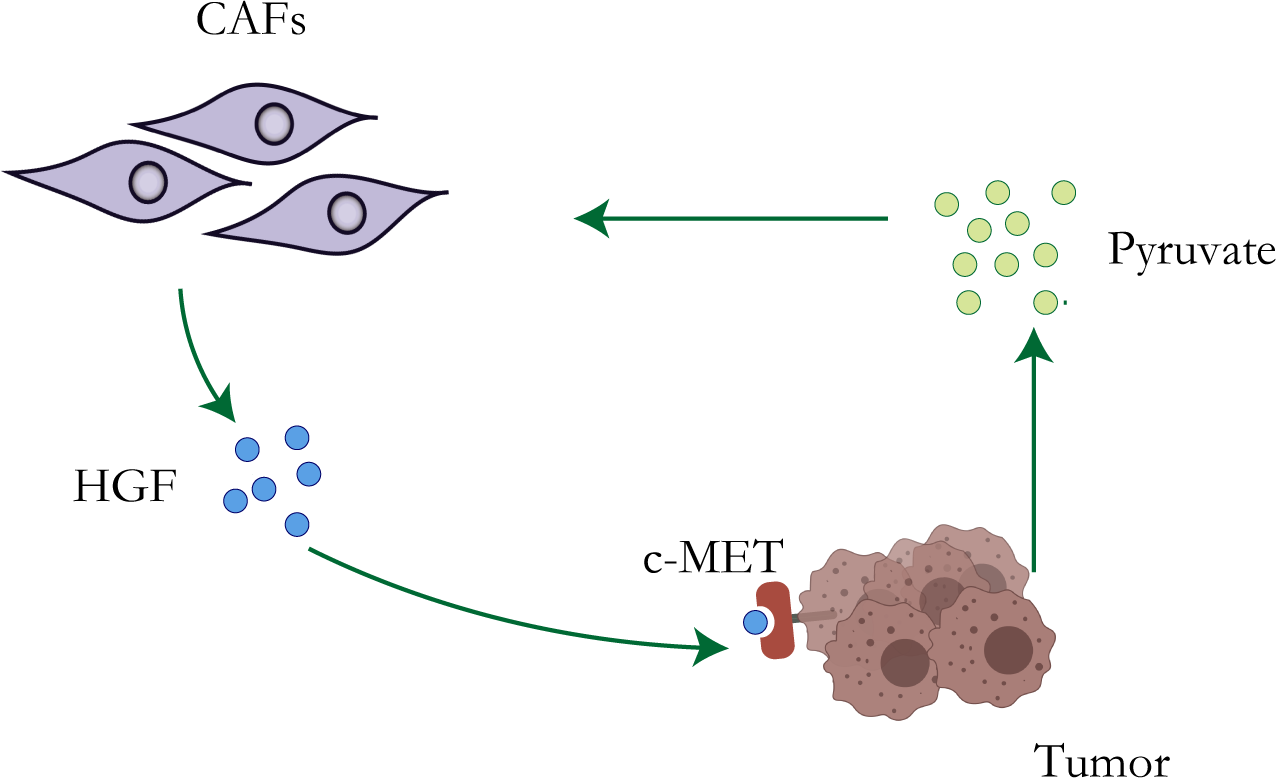
Resistance mechanisms of CAFs. Metabolic reprogramming of tumor cells leads to increased lactate secretion, which stimulates CAFs to up-regulate the production of HGF, leading to chemotherapy resistance of tumor cells.

Although the Warburg effect has been widely accepted as a distinctive character of tumor cells, accumulating evidence has revealed that other metabolic features, particularly the reverse Warburg effect (41), metabolic symbiosis, and glutamine metabolism, create challenges to antitumor therapy due to adaptive or acquired chemotherapy resistance (42). Lisanti et al. proposed the “reverse Warburg effect” in 2009: cancer cells induce glycolysis in cancer-associated fibroblasts which in turn produce lactate and pyruvate that are used by adjacent epithelial cancer cells as sources of the mitochondrial tricarboxylic acid cycle (TCA cycle), oxidative phosphorylation, and ATP production (43, 44) (**Figure 1**). The study of Migneco et al. showed that nutrients derived from glycolytic cancer-associated fibroblasts can effectively reduce the dependence of cancer cells on vascular blood supply, thus promoting the escape of tumor cells during antiangiogenic drugs (45).

In CAA, epidermal growth factor receptor (EGFR) overexpression often indicates a poor prognosis (46). It can be activated by heparin-binding epidermal growth factor (HB-EGF) secreted by CAFs. The HB-EGF/EGFR axis promotes CCA cell proliferation, migration, and invasion by activating the signal transducer and activator of transcription (STAT)-3. Interestingly, TGF-β1 secreted by tumor cells can enhance HB-EGF expression in CAFs, and TGF-β1 production is triggered by EGFR activation. Thus forming a continuous signaling loop (47).

In addition, c-Met expressed by tumor cells in CCA can be activated by HGF secreted by CAFs. After c-Met activation, its downstream pathways including mitogen-activated protein kinase (MAPK), extracellular signal-related kinase (ERK), and phosphatidylinositol 3-kinase (PI3K) are activated, which is conducive to tumor cells invasion and chemotherapy resistance (48, 49).

## Mesenchymal stem cell

Mesenchymal stem cells also called mesenchymal stromal cells(MSCs), migrate to sites of inflammation, as well as migrate and fuse with tumors (50). MSCs are originally isolated from bone marrow. Besides, adipose tissue, peripheral blood, and umbilical cord are also important sources of MSCs (51). MSCs are a heterogeneous subpopulation of progenitor cells with the capability of self-renewal and multidirectional differentiation, which can transdifferentiate into osteocytes, chondrocytes, adipocytes, astrocytes, fibroblasts, and pericytes (52). Studies have shown that MSCs play a significant role in tumorigenesis and development, and participate in many steps of tumorigenesis, such as angiogenesis, invasion, metastasis, epithelial-mesenchymal transformation, anti-apoptosis, immunosuppression, and chemotherapeutic resistance (53). There is growing evidence showing the important role of MSCs in tumor resistance (54).

**Figure 2:**
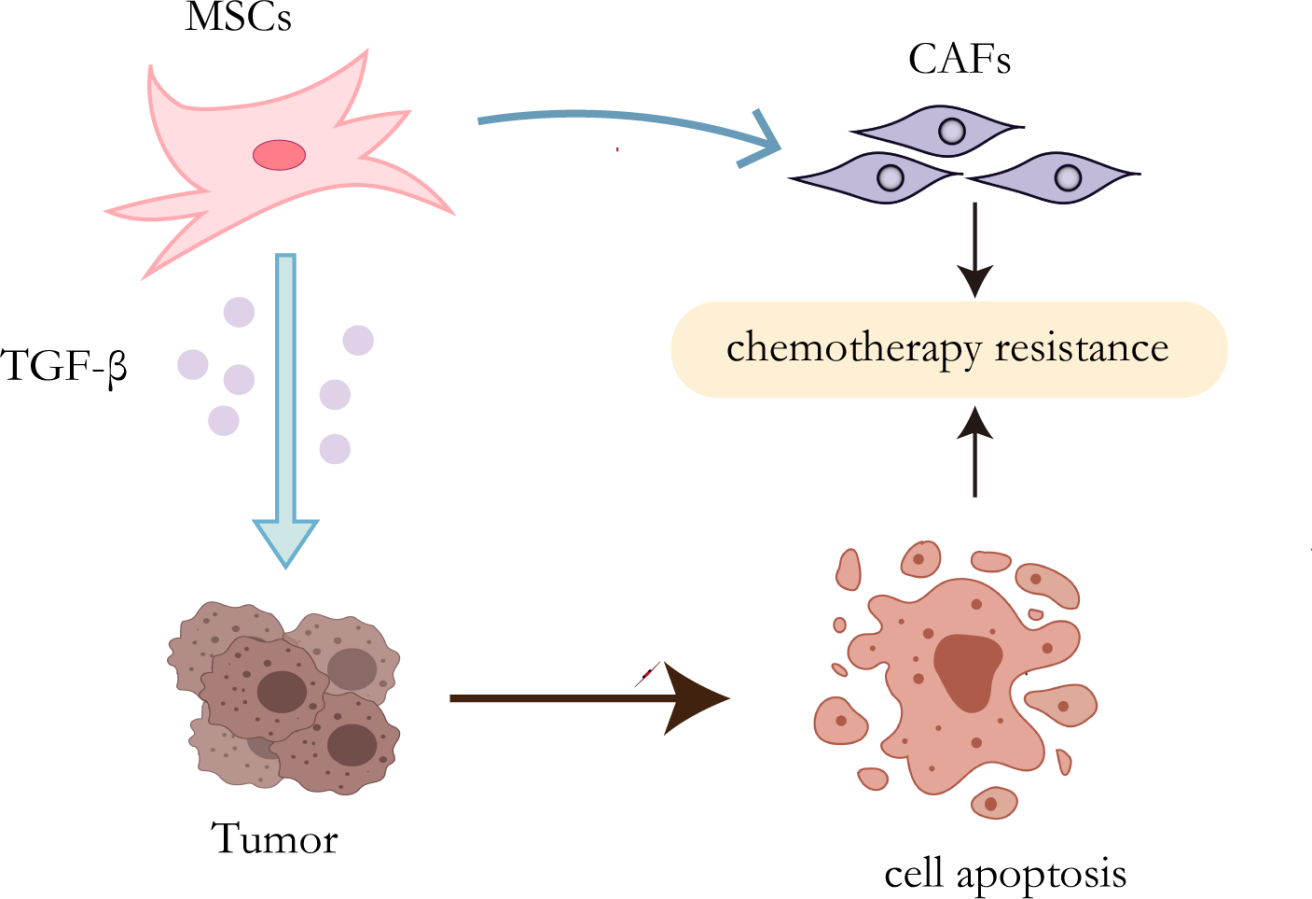
Resistance mechanisms of MSCs. On the one hand, TGF-β secreted by MSCs can induce autophagy of HCC cells, leading to chemotherapy resistance; on the other hand, MSCs can transform into CAFs to promote drug resistance.

It has been proven that MSCs can secrete a variety of cytokines or growth factors that participate in multiple pathways and downstream mechanisms to activate a cascade of reactions, leading to drug resistance (55, 56). Inflammatory mediators are known to be an important part of the tumor microenvironment (57). TGF-β expressed by mesenchymal stem cells increased when exposed to tumor inflammatory microenvironment (58). It has been proved that autophagy can enhance the resistance of HCC cells to chemical drugs by affecting the apoptotic potential of HCC cells (59). Besides, Some studies have indicated that TGF-β plays an important role in inducing autophagy (60, 61). Taken together, MSCs promote the development of chemotherapy resistance of HCC cells by up-regulating the expression of TGF-β (62). MSCs, as mentioned before, have the potential to differentiate into multicellular lineages, so their transformation into CAFs may be another mechanism for the development of therapeutic resistance (**Figure 2**). Mishra et al. found that human bone marrow-derived mesenchymal stem cells (hMSCs) can transform into SDF-1-expressing CAFs when exposed to a tumor-conditioned medium (TCM) for a long time (63). As previously mentioned, CAFs play a critical role in tumor drug resistance.

## Tumor-associated macrophages

Massive macrophage infiltration is a common characteristic of malignant tumors. Based on surface receptors and functional characteristics, macrophages can be divided into classical (M1) and alternative (M2) activated phenotypes. The macrophages surrounding the tumor area are called tumor-associated macrophages (TAM). Increased TAMs are known to be associated with poor prognosis after the surgical resection of hepatocellular carcinoma (64). The majority of macrophages located in intratumoral and peritumoral regions exhibit the M2 phenotype, and several studies have shown that cytokines found in peritumoral tissues, including vascular endothelial growth factor, macrophage colony-stimulating factor, and placental growth factor are significantly associated with HCC recurrence and poor survival outcomes (65–67).

## Cancer-associated adipocytes

Epidemiological evidence suggests that obesity is associated with a combination of all cancers and a poor prognosis for multiple location-specific cancers (68). Systemic and local environmental changes caused by obesity can not only affect the occurrence and development of tumors but also induce chemotherapy resistance to tumors, especially in breast cancer, prostate cancer, ovarian cancer, and leukemia (69, 70). All of this suggests a potential role for adipocytes in tumor drug resistance. Adipocytes are one of the most important components of stromal cells in TME (71). It is noteworthy that co-cultured with cancer cells, adipocytes are observed to exhibit remarkable phenotypic changes and also exhibit an altered phenotype in terms of delipidation, which are designated as cancer-associated adipocytes (CAAs) (72). Bochet et al. demonstrated that tumor cells can secrete Wnt3a to promote changes in adipocyte phenotypes (73). In addition, the bidirectional cross-talk established between cancer cells and mature adipocytes may enhance the invasive capabilities of cancer cells by altering the adipocyte phenotype (72, 74). Accumulating recent evidence indicates that CAAs can induce chemoresistance through different mechanisms such as metabolic reprogramming, secretion of various factors, remodeling of the extracellular matrix, and altering chemotherapy pharmacokinetics (75–78).

**Figure 3:**
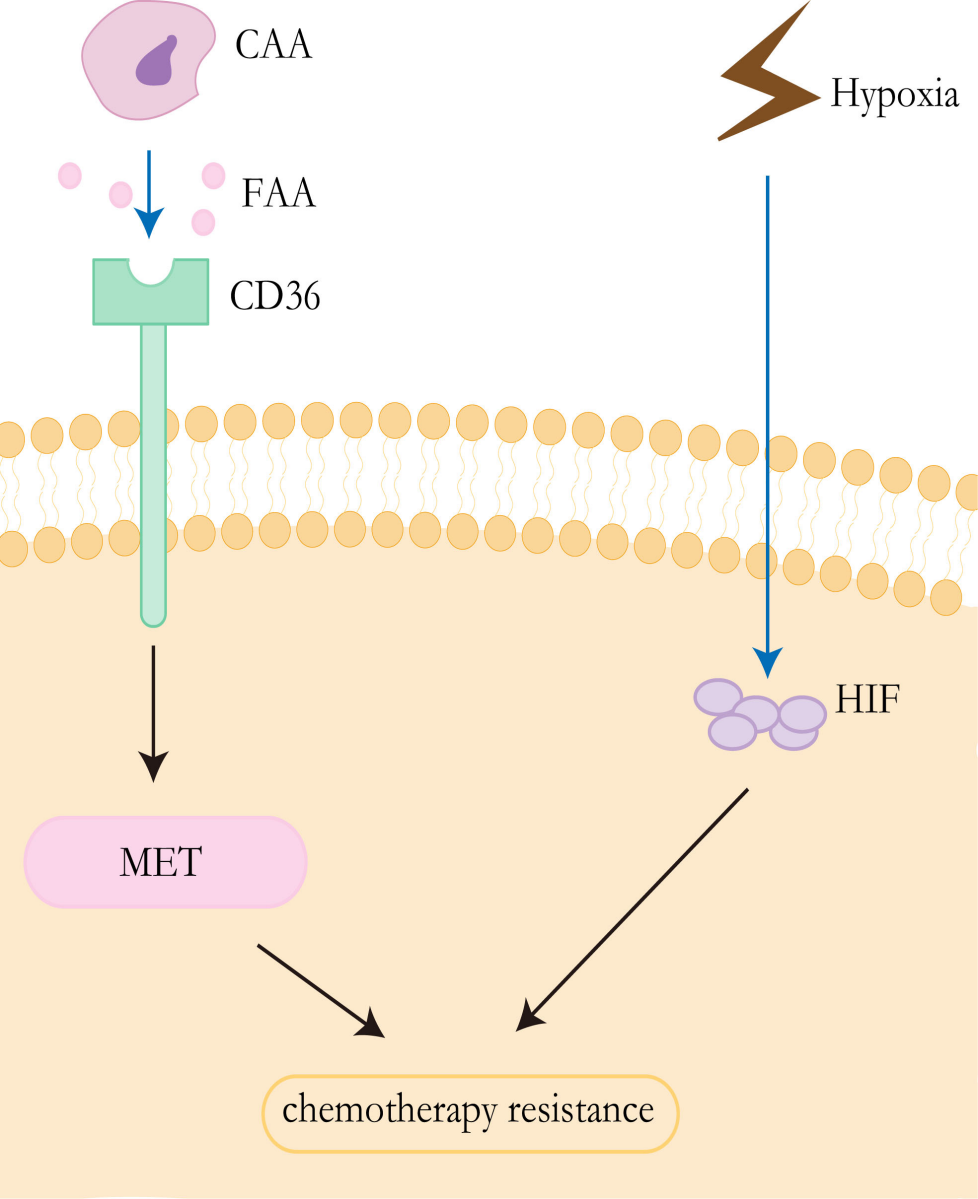
Resistance mechanisms of CAAs and hypoxia. CAAs release exogenous free fatty acids, which are absorbed by CD36 on the surface of tumor cells, to promote epithelial mesenchymal transformation and promote drug resistance of HCC. Tumor cells in anoxic environment lead to upregulated EXPRESSION of HIF, thus promoting drug resistance to chemotherapy.

The “Warburg effect” and “reverse Warburg effect” have been proved to be the cause of CAFs-mediated drug resistance, and this concept can also be applied to other cells in the tumor microenvironment, particularly adipocytes (79). For a long time, adipocytes were identified as a tremendous passive energy storage depot. Pérez de Heredia et al. showed that the release of lactate from adipocytes increased under hypoxic conditions (80). Previous studies have shown that lipids in adipocytes are the main source of tumor cells. CAAs release exogenous free fatty acids (FFAs) that can be taken up by CD36 on the surface of cancer cells (81). FFAs could yield sufficient energy for cancer cells through FAO (82), which contributes to therapy resistance. It has been proved that CD36 promotes epithelial-mesenchymal transition(EMT) in hepatocellular carcinoma by increased free fatty acid uptake (83). Notably, EMT is closely associated with the development of liver cancer and likely affects therapeutic responsiveness in HCC (84) (**Figure 3**).

## Tumor-associated neutrophils

Traditionally, the recruitment mechanism and function of neutrophils have been studied mainly in inflammation. Neutrophils account for about 50-70% of myeloid-derived white blood cells in patients with infection or inflammation (85). The inflammatory cells in solid tumors are mainly neutrophils, and the intratumoral high density is correlated with lymph node metastasis, tumor stage axis, and tumor grade (86). Increasing clinical evidence shows that the tumor and the tumor microenvironment control neutrophil recruitment and in turn tumor-associated neutrophils (TANs) regulate tumor progression or growth control (87). Inflammatory neutrophils not only can phagocytose bacteria, activate and enhance the immunosuppressive system, but TANs also functions as immunosuppressive cells in tumors (88).

**Figure 4:**
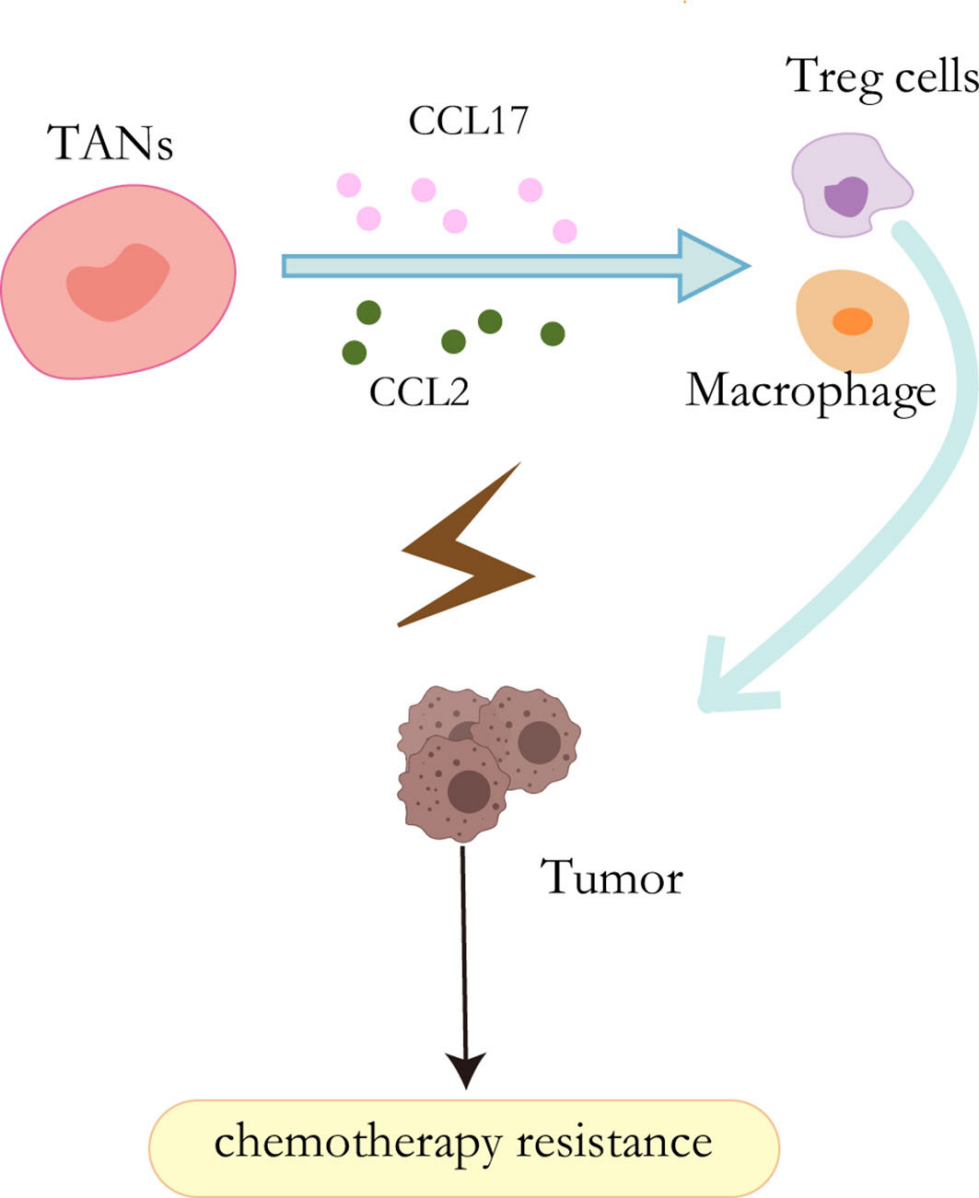
Resistance mechanisms of TANs. CCL2 and CCL17 secreted by TANs can mediate the intratumoral invasion of macrophages and Treg cells, thereby stimulating angiogenesis and promoting sorafenib resistance.

It has been well acknowledged that neutrophils have the capability to secret cytokine and chemokine (89). These cytokines enable them to interact directly with tumor cells as pro-tumor or anti-tumor effectors or indirectly by regulating angiogenesis, tumor growth, and anti-tumor immune responses (90). HGF, a heparin-binding factor, binds to a specific proto-oncogene tyrosine kinase receptor (C-MET) to stimulate hepatocytes by maintaining proliferation, promoting epithelial-mesenchymal transformation (EMT), and ultimately leading to invasion and metastasis during the malignant transformation of HCC (91, 92). In addition, other studies have shown that the HGF-Met axis plays an important role in chemotherapy resistance by coordinating liver cancer metabolism and autophagy. The combination of MET inhibitors and autophagy inhibitors significantly improved the efficacy of hepatocellular carcinoma therapy in mice (93). In liver cancer, however, cancer cells stimulate neutrophils to release hepatocyte growth factors, which in turn stimulates tumor cells to become more aggressive (94). CCL2 and CCL17 secreted by TANs can mediate intratumoral invasion of macrophages and Treg cells, thereby stimulating angiogenesis, promoting liver cancer growth and metastasis, and promoting sorafenib drug resistance. In addition, sorafenib also blocks tumor angiogenesis through targeted VEGF and PDGF receptor kinase activity, which also contributes to TAN accumulation and accompanying macrophage and Treg cell infiltration and promotes sorafenib resistance (95) (**Figure 4**).

## Extracellular matrix

Extracellular matrix (ECM), produced by stromal cells in the microenvironment, provides biochemical and mechanical clues to tumor cells and the surrounding tumor microenvironment. It is a highly dynamic structure that exists in all tissues and is constantly undergoing controlled remodeling. ECM remodeling is essential for regulating tissue morphogenesis (96). It has been demonstrated that some ECM components, directly and indirectly, interact with HCC and stromal cell types to alter the phenotype and function of HCC and stromal cells (97). These mechanical stresses and associated cellular strains could come from externally applied “outside-in” mechanical stimuli. To coordinate with outside-in stimuli, anchored cells also pull on the ECM by increasing ECM adhesions and focal adhesions, i.e. “inside-out” mechanical stimulation (98). This mutual stimulation results in a mechanically rigid microenvironment. The mechanical properties (stiffness) of the tumor cell niche affect both the differentiation of tumor cells and the characteristics of cancer stem cells. It has been shown that increased matrix hardness promotes tumor cell proliferation and chemotherapy resistance (99, 100). Therefore, ECM remodeling plays a crucial role in promoting tumor progression.

## Hypoxia

Anti-angiogenic drugs can cause the tumor’s blood vessels to constrict, resulting in reduced blood flow. Inadequate blood supply affects both the effective delivery of antitumor drugs and the local concentration of oxygen and other nutrients (101). Hypoxia is a common feature of solid tumors, which has been demonstrated to be associated with chemotherapy failure and plays an important role in the selection of more aggressive and resistant clones, and poor clinical outcomes (102–104). The viability of hypoxic cells in solid tumors is increased by the adaptive response of cells to hypoxia, which is mainly controlled by hypoxia-inducible factors (HIFs) (105, 106). HIFs are transcription factors that mediate the adaptation of tumor cells to hypoxia by regulating genes involved in cell proliferation, angiogenesis, glucose metabolism, tumor invasion, and metastasis (103, 106). The HIFs factor consists of the HIF-α subunit and HIF-β subunit, where the α-subunit includes three subtypes(HIF-1α, HIF-2α, and HIF-3α) (107). Overexpression of HIF-1α and HIF-2α has been detected in nonalcoholic fatty liver disease, HCC, alcoholic liver disease, and radioactive liver injury (106). Studies have demonstrated that the multidrug resistance 1(MDR1/ABCB1) gene is hypoxia reactive and that HIF-1α can lead to the activation of the gene (108). Multidrug resistance (MDR1) gene product P-glycoprotein can reduce the intracellular concentration of sorafenib and other drugs, which is related to chemotherapy resistance (109). In addition, the stabilization of HIF1α can lead to metabolic reprogramming and enhance tumor growth (110) (**Figure 3**).

## Conclusion and future development

The overall treatment outcome for liver cancer is far from satisfactory. The use of chemotherapeutic drugs has been hampered by drug resistance mechanisms in which the tumor microenvironment is an indispensable player. TME is a dynamic and constantly changing complex biological network whose diverse cellular and non-cellular components externally influence hallmarks and fates of tumor cells, which in part contributes to resistance to conventional therapeutic drugs. Therefore, TME would be an attractive target, both to sensitize tumors to traditional therapies and as a new option to fight the disease. Anyway, our current insights into tumor therapy indicate that the rapid elimination of therapeutic resistance in tumor cells is critical to reducing the incidence of adverse events. Although different combinations of therapies promise to achieve this goal, it is critical to find novel strategies to block primary crosstalk and reshape the microenvironment. It’s believed that in the foreseeable future, more and more preclinical and clinical research is expected to translate into novel, effective and safe clinical treatment options.

## Author contributions

ZY and ZR designed the study. CZ, SL, FG, and YZ collected data and summarized the viewpoints. CZ and SL analyzed data and draw the figures. CZ wrote the manuscript. All authors reviewed and approved the manuscript.

## Funding

This study was sponsored by grants from National Natural Science Foundation of China (U2004121, 82070643 and U1904164), and Research Project of Jinan Microecological Biomedicine Shandong Laboratory (JNL-2022015B).

## Conflict of interest

The authors declare that the research was conducted in the absence of any commercial or financial relationships that could be construed as a potential conflict of interest.

## Publisher’s note

All claims expressed in this article are solely those of the authors and do not necessarily represent those of their affiliated organizations, or those of the publisher, the editors and the reviewers. Any product that may be evaluated in this article, or claim that may be made by its manufacturer, is not guaranteed or endorsed by the publisher.
